# Combination of a Selective HSP90α/β Inhibitor and a RAS-RAF-MEK-ERK Signaling Pathway Inhibitor Triggers Synergistic Cytotoxicity in Multiple Myeloma Cells

**DOI:** 10.1371/journal.pone.0143847

**Published:** 2015-12-02

**Authors:** Rikio Suzuki, Shohei Kikuchi, Takeshi Harada, Naoya Mimura, Jiro Minami, Hiroto Ohguchi, Yasuhiro Yoshida, Morihiko Sagawa, Gullu Gorgun, Diana Cirstea, Francesca Cottini, Jana Jakubikova, Yu-Tzu Tai, Dharminder Chauhan, Paul G. Richardson, Nikhil Munshi, Kiyoshi Ando, Teruhiro Utsugi, Teru Hideshima, Kenneth C. Anderson

**Affiliations:** 1 Jerome Lipper Multiple Myeloma Center, Department of Medical Oncology, Dana-Farber Cancer Institute, Harvard Medical School, Boston, MA, United States of America; 2 Department of Hematology/Oncology, Tokai University School of Medicine, Isehara, Kanagawa, Japan; 3 Tsukuba Research Center, Taiho Pharmaceutical Co., Ltd., Tsukuba, Japan; 4 VA Boston Healthcare System, Jerome Lipper Multiple Myeloma Center, Dana-Farber Cancer Institute, Harvard Medical School, Boston, MA, United States of America; University of Queensland Diamantina Institute, AUSTRALIA

## Abstract

Heat shock protein (HSP)90 inhibitors have shown significant anti-tumor activities in preclinical settings in both solid and hematological tumors. We previously reported that the novel, orally available HSP90α/β inhibitor TAS-116 shows significant anti-MM activities. In this study, we further examined the combination effect of TAS-116 with a RAS-RAF-MEK-ERK signaling pathway inhibitor in *RAS*- or *BRAF*-mutated MM cell lines. TAS-116 monotherapy significantly inhibited growth of *RAS*-mutated MM cell lines and was associated with decreased expression of downstream target proteins of the RAS-RAF-MEK-ERK signaling pathway. Moreover, TAS-116 showed synergistic growth inhibitory effects with the farnesyltransferase inhibitor tipifarnib, the BRAF inhibitor dabrafenib, and the MEK inhibitor selumetinib. Importantly, treatment with these inhibitors paradoxically enhanced p-C-Raf, p-MEK, and p-ERK activity, which was abrogated by TAS-116. TAS-116 also enhanced dabrafenib-induced MM cytotoxicity associated with mitochondrial damage-induced apoptosis, even in the *BRAF*-mutated U266 MM cell line. This enhanced apoptosis in *RAS*-mutated MM triggered by combination treatment was observed even in the presence of bone marrow stromal cells. Taken together, our results provide the rationale for novel combination treatment with HSP90α/β inhibitor and RAS-RAF-MEK-ERK signaling pathway inhibitors to improve outcomes in patients with in *RAS-* or *BRAF-*mutated MM.

## Introduction

Multiple myeloma (MM) is characterized by proliferation of clonal plasma cells in the bone marrow (BM) microenvironment, monoclonal protein in the blood and/or urine, bone lesions, and immunodeficiency [1,2]. In recent years, the introduction of high-dose chemotherapy and stem cell transplantation, as well as novel therapies including bortezomib, thalidomide, and lenalidomide, have prolonged the survival of patients with MM [1,3]. However, relapses are common, and novel therapies are needed.

An inhibitor of heat shock protein (HSP)90 is a promising novel targeted therapy [4]. Since the prototype geldanamycin was first isolated in 1970 from *Streptomyces higroscopicus var geldanus*, HSP90 inhibitors have been developed as treatment options for specific molecularly-defined subgroups of cancer [5]. Most HSP90 inhibitors bind to the ATP-binding sites in the N-terminal domain of HSP90, thereby inhibiting the interaction between HSP90 and client proteins as well as the folding and maturation of client proteins, leading to the destruction of client proteins via the ubiquitin-proteasome system [5,6]. Compared with normal cells, malignant cells including MM cells are highly dependent on HSP90 systems to overcome cellular stress induced by abnormal fusion proteins and/or a hypoxic, acidotic, and nutrient-deprived microenvironment [6–9]. Moreover, in malignant tissues HSP90 forms specific multi-chaperone complexes, which have higher affinity for oncoproteins than HSP90 in normal tissues [5,10]. Thus, HSP90 is an attractive molecular target for cancer therapy.

Since first-generation HSP90 inhibitors have shown various toxicities, i.e. a geldanamycin analog causes liver toxicity with poor solubility [11,12], second-generation HSP90 inhibitors have been developed and are currently in clinical trials. However, the effectiveness of some HSP90 inhibitor monotherapies may be limited in the clinical setting [4,5,13]. Moreover, HSP90 inhibitor SNX-5422-induced ocular toxicity has been observed, resulting in discontinuation of its clinical evaluation [5,14]. Thus, ongoing efforts are developing less toxic, second-generation HSP90 inhibitors and identifying biomarkers algorithm to identify the most appropriate patient populations to benefit.

Our group reported that several HSP90 inhibitors (17-AAG, SNX-2112, and TAS-116) show promising anti-MM effects [7,15,16]. TAS-116 is an orally active, ATP competitive inhibitor of HSP90α/β [17,18]. In particular, TAS-116 shows favorable pharmacokinetics and a reduced ocular toxicity profile, possibly due to its lower distribution in retinal tissue than in plasma in rats [17]. Moreover, TAS-116 shows superior anti-tumor effects in several tumors including MM and lung carcinoma *in vitro* and *in vivo* [16,17]. Therefore, TAS-116 represents a promising therapeutic potential.

The rat sarcoma (RAS)-v-raf murine sarcoma viral oncogene homolog (RAF)-mitogen-activated protein kinase/extracellular signal-regulated kinase kinase (MEK)-extracellular signal-regulated kinase (ERK) signaling pathway is one of the most important oncogenic pathways which plays a central role in regulation of cell proliferation and survival [19]. Aberrant signaling through this pathway is common in a wide variety of malignancies, including MM, making it an attractive candidate for development of novel targeted therapies [20]. Many cytokines (i.e., interleukin (IL)-6, insulin-like growth factor-1, stromal cell derived factor-1α (SDF1α), and BAFF (B cell activating factor)) activate the RAS-RAF-MEK-ERK signaling cascade and mediate MM cell proliferation [21,22].

A recognized genetic difference between monoclonal gammopathy of undetermined significance (MGUS) and MM is *RAS* mutation, which is extremely rare in MGUS but present in 20–30% of newly diagnosed MM [23]. The RAS pathway plays a main role in switching of MGUS to MM, since activating *RAS* mutations (mainly *neuroblastoma ras viral oncogene homolog (NRAS)* or *v-ki-ras2 kirsten rat sarcoma viral oncogene homolog (KRAS)*) are found in 32–50% of patients with MM [20]. Our group and others have previously reported that *RAS* mutation is an independent prognostic factor in MM [24], and that *NRAS* mutation significantly reduces MM sensitivity to single-agent bortezomib therapy [25]. Many RAS pathway inhibitors, including RAF inhibitors and MEK inhibitors, have been developed and show superior effects in the treatment of malignant melanoma, Her2-positive breast cancer, and anaplastic lymphoma kinase (ALK)-positive NSCLC [19]. However, RAF inhibitors and MEK inhibitors essentially produce a cytostatic effect and show limited efficacy as a monotherapy [20]. Therefore, a second type of therapy that synergizes with the anti-tumor effects of RAF or MEK inhibitors is needed. Recently, some groups have reported that the combination of RAF inhibitors and MEK inhibitors shows significant synergistic anti-tumor effects in melanoma with v-raf murine sarcoma viral oncogene homolog B1 (BRAF) V600E mutation [26,27]. However, dabrafenib shows paradoxical effects, in which proliferation of tumors harboring wild-type *RAF* and *RAS* mutation is promoted rather than inhibited [28]. Moreover, acquisition of resistance to dabrafenib has recently been described [29,30]. Therefore, an optimal partner that overcomes these resistance mechanisms is needed.

Another group reported that the combination of ganetespib with MEK inhibitors shows significant synergistic anti-tumor effects against NSCLCs with *RAS* mutations *in vitro* and *in vivo* [31]. In the present study, we demonstrate that TAS-116 in combination with an inhibitor of the RAS-RAF-MEK-ERK signaling pathway shows significant synergistic anti-myeloma effects in *RAS*- or *BRAF*-mutated MM cell lines *in vitro*, providing the framework for its clinical evaluation to improve MM patient outcome.

## Methods

### Reagents

The HSP90 inhibitor TAS-116 was synthesized at Taiho Pharmaceutical Co., Ltd. (Tsukuba, Japan). Bortezomib, doxorubicin, tipifarnib, dabrafenib, and AZD6244 were obtained from Selleck Chemicals (Houston, TX, USA). Recombinant human IL-6 was from R&D Systems (Minneapolis, MN, USA).

### Human cell lines

The dexamethasone (Dex)-sensitive MM.1S MM cell line was kindly provided by Dr. Steven Rosen (Northwestern University, Chicago, IL, USA). RPMI-8226, U266, and NCI-H929 human MM cell lines were obtained from ATCC (Manassas, VA, USA). The doxorubicin (Dox)-resistant RPMI-8226 DOX40 cell line was kindly provided by Dr. William Dalton (Lee Moffitt Cancer Center, Tampa, FL, USA). The IL-6-dependent INA6 human cell line was provided by Dr. Renate Burger (University of Kiel, Kiel, Germany). KMS-11 was kindly provided by Dr. Takemi Otsuki (Kawasaki Medical School, Okayama, Japan). All MM cell lines were cultured in RPMI 1640 containing 10% FBS (Sigma-Aldrich, St Louis, MO, USA), 2 μM l-glutamine, 100 U/mL penicillin, and 100 μg/mL streptomycin (Invitrogen, Carlsbad, CA, USA), plus 2.5 ng/mL IL-6 only for INA6 cells.

### Primary cells

Patient MM cells and bone marrow stromal cells (BMSCs) were obtained from BM samples after informed consent was obtained. All participants provided their written informed consent to participate in this study. This study was performed in accordance with the Declaration of Helsinki and was approved by the Institutional Review Board of the Dana-Farber Cancer Institute. Mononuclear cells were separated using Ficoll-Hypaque density sedimentation, and plasma cells were purified (>95% CD138^+^) by positive selection with anti-CD138 magnetic-activated cell separation microbeads (Miltenyi Biotec, San Diego, CA, USA). Tumor cells were also purified from the BM of MM patients using the RosetteSep negative selection system (StemCell Technologies, Vancouver, BC, Canada). BMSCs were generated by culturing BM mononuclear cells for 4 to 6 weeks in DMEM supplemented with 15% FBS, 100 U/mL penicillin, and 100 μg/mL streptomycin.

### Growth inhibition assay

The growth inhibitory effect of TAS-116 or RAS-RAF-MEK-ERK inhibitors in MM cell lines was assessed by measuring 3-(4,5-dimethylthiazol-2-yl)-2,5-diphenyl tetrasodium bromide (MTT; Sigma-Aldrich) dye absorbance as described [32].

### Western blotting

MM cells were treated with or without novel or conventional agents, harvested, washed, and lysed as reported [32,33]. Cell lysates were subjected to SDS-PAGE, transferred to membranes, and immunoblotted with the following antibodies: anti-p27, cyclin D1, phospho-B-Raf (Ser445), B-Raf, phospho-v-raf-1 murine leukemia viral oncogene homolog 1 (C-Raf) (Ser338), C-Raf, phospho-MEK1/2 (Ser217/221), MEK1/2, phospho-ERK (Thr202/Tyr204), ERK, phospho-v-akt murine thymoma viral oncogene homolog (Akt) (Ser473), Akt, caspase 3, poly (ADP-ribose) polymerase (PARP), β-actin, Bim, α-tubulin (all from Cell Signaling, Beverly, MA, USA), as well as NRAS and KRAS (both from Santa Cruz Biotechnology, Dallas, TX, USA).

### Transient transfection

INA6 cells were transiently transfected with non-targeting short interfering RNA (siRNA) or *NRAS* siRNA siGENOME SMARTpool siRNA (Dharmacon, Inc., Lafayette, CO, USA). RPMI-8226 and RPMI-8226 DOX40 cells were transiently transfected with non-targeting siRNA or *KRAS* siRNA siGENOME SMARTpool siRNA (Dharmacon) using Nucleofector Kit V (Amaxa Biosystems, Cologne, Germany). Cells were harvested 24–72 h after transfection and analyzed with immunoblotting and the cell viability assay.

### Detection of apoptosis with annexin V/propidium iodide (PI) staining

Detection of apoptotic cells was done with the annexin V/ PI detection kit (Immunotech/Beckman Coulter, Indianapolis, IN, USA) as described [34]. Apoptotic cells were analyzed on a BD FACSCanto II (BD Biosciences) using FACSDiva (BD Biosciences). Cells that were annexin V positive and PI negative were considered early apoptotic cells, whereas positivity for both annexin V and PI was associated with late apoptosis or necrosis.

### Mitochondrial membrane potential

To evaluate the effect of TAS-116 on alterations of mitochondrial membrane potential, MM cells were treated with or without novel or conventional agents with addition of MitoCapture reagent (MitoCapture Apoptosis Detection kit®, Calbiochem) for the last 20 minutes, followed by flow cytometric analysis on a BD FACSCanto II (BD Biosciences) using FACSDiva® (BD Biosciences) [35].

### Statistical analysis

Statistical significance was determined with the Student’s t-test. The minimal level of significance was *P* < 0.05. The combination index (CI) values were calculated by isobologram analysis using the CompuSyn Version 1.0 software program (ComboSyn, Paramus, NJ, USA). CI < 1.0 indicates synergism; CI = 1.0 indicates an additive effect; and CI > 1.0 indicates antagonism.

## Results

### Downregulation of RAS inhibits growth and enhances cytotoxicity of doxorubicin and bortezomib in *RAS*-mutated MM cell lines

**Fig 1:**
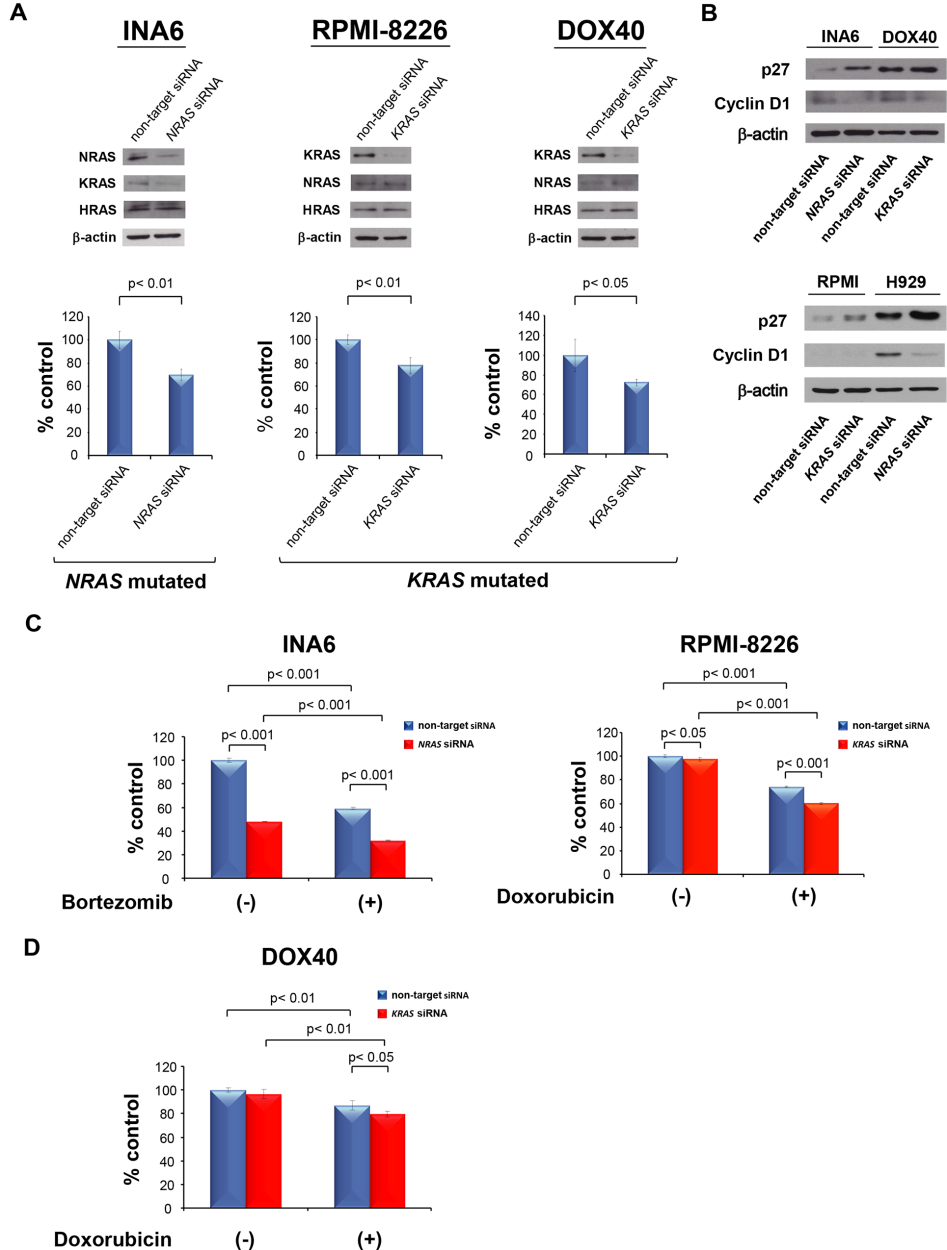
Downregulation of RAS inhibits growth and enhances cytotoxicity of doxorubicin and bortezomib in *RAS*-mutated MM cell lines. (A) INA6 cells were transiently transfected with non-targeting or *NRAS* siRNA, and RPMI-8226 and RPMI-8226 DOX40 cells were transiently transfected with non-targeting or *KRAS* siRNA. The cell number and viability 48 h later were assessed with trypan blue exclusion. Whole-cell lysates were subjected to western blotting to confirm the downregulation of NRAS and KRAS expression using NRAS, KRAS, HRAS, and β-actin Abs. Data are the mean ± SD of triplicate wells. (B) INA6 and NCI-H929 cells were transiently transfected with non-targeting or *NRAS* siRNA, and RPMI-8226 and RPMI-8226 DOX40 cells were transiently transfected with non-targeting or *KRAS* siRNA. After 48 h, whole-cell lysates were subjected to western blotting using p27, cyclin D1, NRAS and β-actin Abs. (C) INA6 cells were transiently transfected with non-targeting or *NRAS* siRNA and then treated with or without bortezomib (5 nM) for 48 h. RPMI-8226 cells were transiently transfected with non-targeting or *KRAS* siRNA and then treated with or without doxorubicin (0.1 μM) for 48 h. In each case, cell viability was assessed with the MTT assay of triplicate cultures and expressed as the percentage of the untreated control. Data are the mean ± SD. (D) RPMI-8226 DOX40 cells were transiently transfected with non-targeting or *KRAS* siRNA and then treated with or without doxorubicin (1 μM) for 24 h. Cell viability was assessed with the MTT assay of triplicate cultures and expressed as the percentage of the untreated control. Data are the mean ± SD.

First, we assessed the functional significance of NRAS and KRAS in *RAS*-mutated MM cells using a siRNA strategy. The viability of *NRAS*-mutated INA6 cells was markedly inhibited by *NRAS* siRNA compared with non-targeting siRNA and was associated with significant downregulation of NRAS expression. Similarly, the viability of *KRAS*-mutated RPMI-8226 and RPMI-8226 DOX40 cells was inhibited by *KRAS* siRNA compared with non-targeting siRNA, associated with significant downregulation of KRAS expression (Fig 1A).

Others have shown that the RAS-RAF-MEK-ERK signaling pathway is necessary for G1 progression and that it also regulates cyclin D1 transcription and p27 expression [36–38]. We therefore hypothesized that *RAS* siRNA-induced growth inhibition may be associated with cell cycle arrest. Indeed, we observed downregulation of cyclin D1 and upregulation of p27 in both *NRAS* siRNA-transfected INA6 and NCI-H929 as well as *KRAS* siRNA-transfected RPMI-8226 and RPMI-8226 DOX40 cells, compared with non-targeting siRNA-transfected cells (Fig 1B). These results suggest that downregulation of RAS in *RAS*-mutated MM cells regulates MM cell survival, at least in part, via cell cycle modulation.

We and others have previously shown that *NRAS* or *KRAS* mutation contributes to resistance to conventional chemotherapy or bortezomib [25,39]. Therefore, we next examined whether RAS inhibition enhances conventional chemotherapy- or bortezomib-induced cytotoxicity in *RAS*-mutated MM cells. Bortezomib- or doxorubicin-triggered cytotoxicity was significantly enhanced in NRAS knockdown INA6 and NCI-H929 cells, but not in KMS11 cells (Fig 1C left and S2 Fig); moreover, cytotoxicity of doxorubicin was also enhanced in KRAS knockdown RPMI-8226 cells and doxorubicin-resistant RPMI-8226 DOX40 cells (Fig 1C right and Fig 1D). These results indicate that RAS inhibition enhances conventional chemotherapy- or bortezomib-induced cytotoxicity in *RAS*-mutated MM cells.

### TAS-116 induces cytotoxicity and targets degradation of the HSP90 client RAS-RAF-MEK-ERK signaling pathway proteins in *RAS*-mutated MM cells

**Fig 2:**
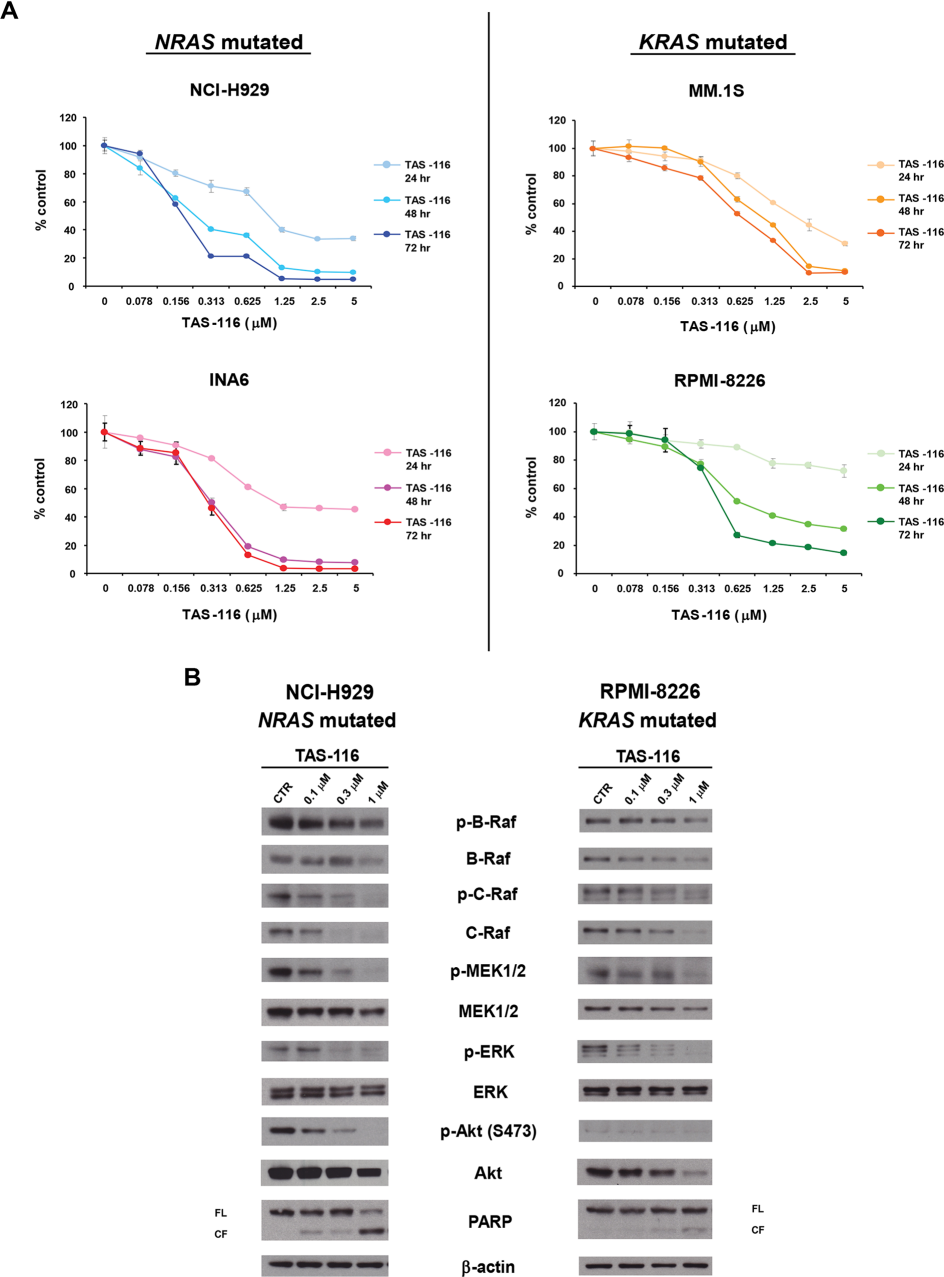
TAS-116 effects on cell viability and RAS-RAF-MEK-ERK signaling in *RAS*-mutated MM cell lines. (A) NCI-H929, INA6, MM.1S, and RPMI-8226 MM cell lines were cultured with TAS-116 (0–5 μM) for 24, 48, or 72 h. In each case, cell viability was assessed with the MTT assay of triplicate cultures and expressed as the percentage of the untreated control. Data are the mean ± SD. (B) NCI-H929 and RPMI-8226 cells were treated with the indicated doses of TAS-116 for 24 h. Whole-cell lysates were subjected to western blotting using p-B-Raf, B-Raf, p-C-Raf, C-Raf, p-MEK1/2, MEK1/2, p-ERK, ERK, p-Akt (S473), Akt, PARP, and β-actin Abs. FL, full-length; CF, cleaved form.

We next examined the cytotoxicity of TAS-116 in a panel of MM cell lines selected for expression of *NRAS* or *KRAS* mutations. TAS-116 is a selective inhibitor of cytoplasmic HSP90α/β that does not inhibit HSP90 paralogs, such as endoplasmic reticulum GRP94 or mitochondrial TRAP1 (S1 Fig). TAS-116 significantly inhibited the viability in all *RAS*-mutated MM cells in a dose- and time-dependent manner (Fig 2A). We also measured expression levels of RAS-RAF-MEK-ERK pathway client proteins following TAS-116 treatment. TAS-116 triggered significant degradation of key RAS-RAF-MEK-ERK pathway regulators (p-C-Raf, p-MEK1/2, and p-ERK) in a dose-dependent manner in *RAS*-mutated MM cell lines (Fig 2B). In these cell lines, especially NCI-H929 cells, we observed that a modest reduction in p-B-Raf, marked induction of PARP cleavage, and inhibition of p-Akt were induced by TAS-116 in a dose-dependent manner (Fig 2B). Furthermore, we confirmed that TAS-116 triggered a decrease in mitochondria transmembrane potential in a time-dependent manner (S3 Fig). Taken together, these results indicate that TAS-116 potently targets HSP90 client RAS-RAF-MEK-ERK signaling pathway proteins, and induces cytotoxicity, associated with mitochondrial alterations and apoptosis in *RAS*-mutated MM cell lines.

### Farnesyltransferase inhibitor and selective RAF or MEK inhibitors trigger cytotoxicity and induce apoptosis in *RAS*-mutated MM cells

**Fig 3:**
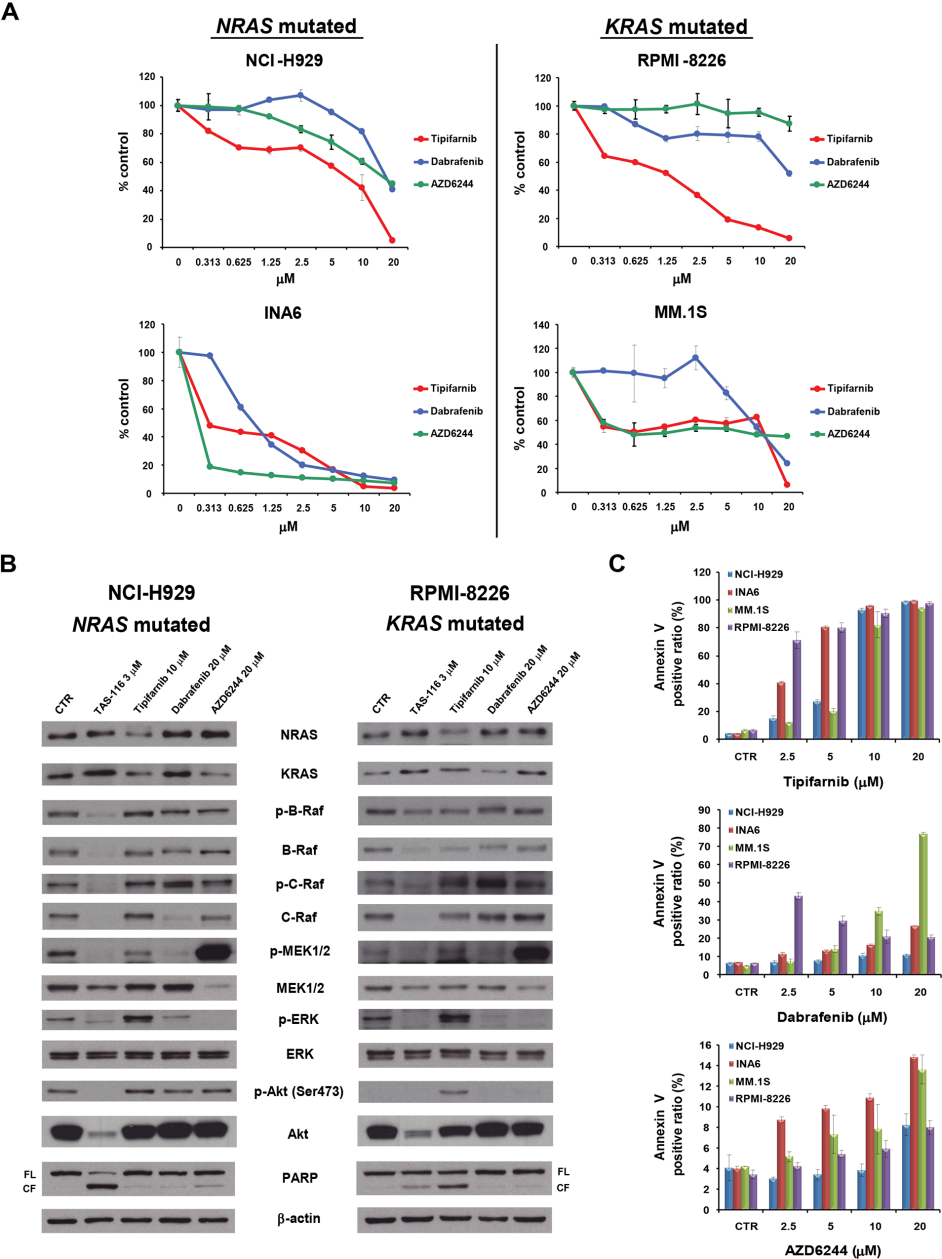
RAS pathway inhibitors induce cytotoxicity and apoptosis in *RAS*-mutated MM cell lines. (A) NCI-H929, INA6, MM.1S, and RPMI-8226 MM cell lines were cultured with tipifarnib (0–20 μM), dabrafenib (0–20 μM), or AZD6244 (0–20 μM) for 72 h. In each case, cell viability was assessed with the MTT assay of triplicate cultures and expressed as the percentage of the untreated control. Data are the mean ± SD. (B) NCI-H929 and RPMI-8226 cells were treated with TAS-116 (3 μM), tipifarnib (10 μM), dabrafenib (20 μM), or AZD6244 (20 μM) for 24 h. Whole-cell lysates were subjected to western blotting using NRAS, KRAS, p-B-Raf, B-Raf, p-C-Raf, C-Raf, p-MEK1/2, MEK1/2, p-ERK, ERK, p-Akt (S473), Akt, PARP, and β-actin Abs. FL, full-length; CF, cleaved form. (C) NCI-H929, INA6, MM.1S, and RPMI-8226 cells were treated with tipifarnib (0–20 μM), dabrafenib (0–20 μM), or AZD6244 (0–20 μM) for 48 h. Apoptotic cells were analyzed with flow cytometry using annexin V/PI staining. Each treatment was tested in triplicate wells, and apoptosis was assessed as the percentage of annexin V-positive cells.

We next examined the cytotoxicity of the farnesyltransferase inhibitor tipifarnib, the selective RAF inhibitor dabrafenib, or the selective MEK inhibitor AZD6244, in *RAS*-mutated MM cell lines. These inhibitors induced modest to moderate cytotoxicity in each *RAS*-mutated MM cell line, with the IL-6 dependent INA6 cells being the most sensitive (Fig 3A left, lower panel). Interestingly, a broad range of tipifarnib and AZD6244 (0.313 μM to 10 μM) induced equivalent cytotoxicity, suggesting cytostatic effects in this cell line (Fig 3A right, lower panel). We next examined the effect of these inhibitors on degradation of RAS-RAF-MEK-ERK signaling pathway client proteins. Tipifarnib inhibited NRAS and KRAS in *NRAS*-mutated NCI-H929 cells, as well as NRAS in *KRAS*-mutated RPMI-8226 cells. However, tipifarnib markedly increased the RAS-RAF-MEK-ERK signaling pathway proteins p-B-Raf, p-C-Raf, and p-ERK in NCI-H929 cells; as well as p-C-Raf, p-MEK1/2, and p-ERK in RPMI-8226 cells. In addition, PARP cleavage and p-Akt (Ser473) were increased in both cell lines, associated with a feedback loop mechanism (Fig 3B). Interestingly, dabrafenib increased NRAS and KRAS in NCI-H929 cells, and NRAS in RPMI-8226 cells. Moreover, dabrafenib increased p-C-Raf and decreased p-MEK1/2 and p-ERK in both cell lines (Fig 3B). Although others have previously shown that RAF inhibitors paradoxically activate ERK signaling in wild-type *BRAF* and *RAS*-mutated tumors [28,40–42], we here confirmed that dabrafenib (1.25 to 2.5 μM) activated p-MEK1/2 and p-ERK in both cell lines, suggesting that paradoxical activation also occurs in MM (S4 Fig).

We next investigated the mechanism of cytotoxicity in *RAS*-mutated MM cells triggered by RAS-RAF-MEK-ERK signaling pathway inhibitors using annexin V/PI staining. We observed a dose-dependent increase in annexin V-positive cells in these *RAS*- mutated MM cell lines after treatment with each inhibitor (*P* < 0.01 in each tipifarnib treatment doses versus control groups in each cell lines; *P* < 0.05 in AZD6244 treatment doses versus control groups in each cell lines; P < 0.05 in 5, 10, and 20 μM dabrafenib treatment doses versus control groups in NCI-H929 and MM.1S cells) (Fig 3C). We also confirmed that these inhibitors induced mild or moderate PARP cleavage in NCI-H929 and RPMI-8226 cells (Fig 3B). Taken together, these results indicate that a farnesyltransferase inhibitor and selective RAF or MEK inhibitors trigger cytotoxicity and induce apoptosis in *RAS*-mutated MM cells.

### The combination of TAS-116 and tipifarnib, dabrafenib, or AZD6244 triggers synergistic anti-MM activity

**Fig 4:**
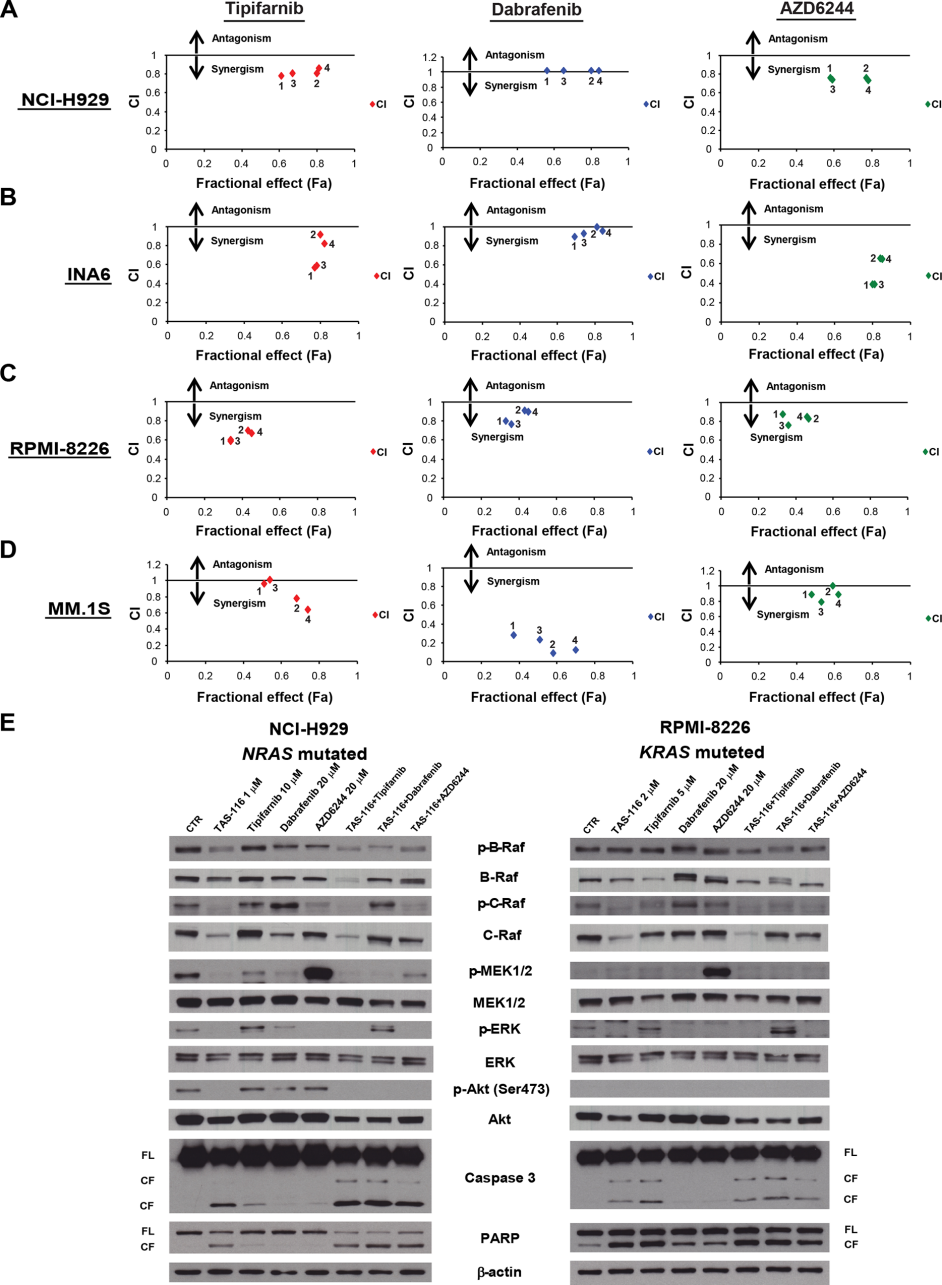
Combination of TAS-116 plus tipifarnib, dabrafenib, or AZD6244 triggers synergistic anti-MM activity. (A) NCI-H929 cells, (B) INA6, (C) RPMI8226, and (D) MM1.S cells were treated with TAS-116 (0–1 μM) in combination with tipifarnib (0–2 μM), dabrafenib (0–5 μM), or AZD6244 (0–4 μM) for 48 h, and then the viability was analyzed with the MTT assay. Isobologram analysis shows the synergistic or additive cytotoxic effect of TAS-116 and each drug. The graphs are derived from the values given in S1 Table (A), S2 Table (B), S3 Table (C), and S4 Table (D). The numbers 1–4 in each graph correspond to the combinations shown in S1 Table. CI values < 1.0 indicate synergism; CI = 1.0 indicate an additive effect; and CI > 1.0 indicates antagonism. (E) NCI-H929 and RPMI-8226 cells were treated with the indicated concentrations of TAS-116 either alone or in combination with tipifarnib, dabrafenib, or AZD6244 for 24 h. Whole-cell lysates were subjected to western blotting using p-B-Raf, B-Raf, p-C-Raf, C-Raf, p-MEK1/2, MEK1/2, p-ERK, ERK, p-Akt (S473), Akt, caspase 3, PARP, and β-actin Abs. FL, full-length; CF, cleaved form.

**Fig 5:**
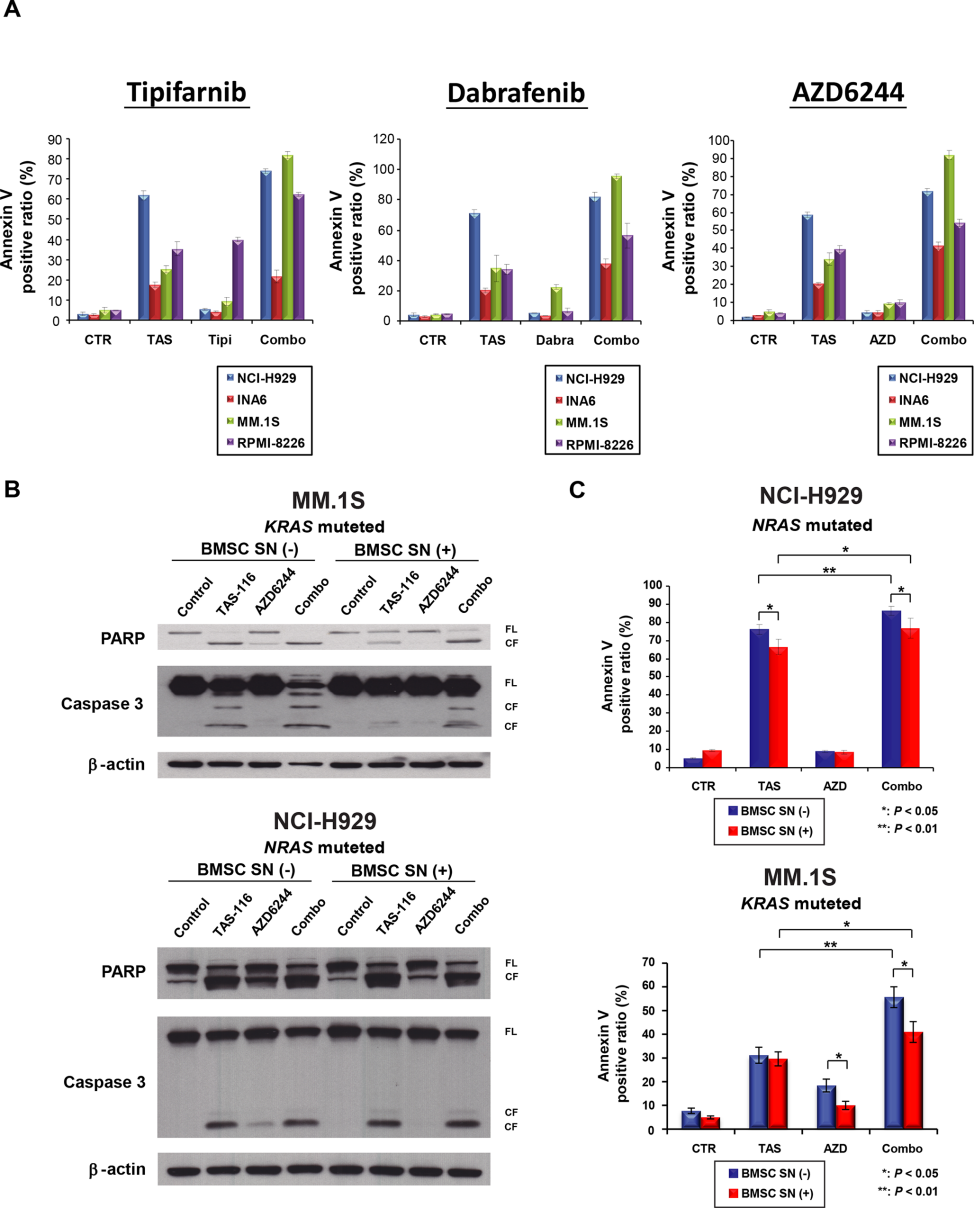
Combination of TAS-116 plus tipifarnib, dabrafenib, or AZD6244 blocks the growth stimulatory effect of the bone marrow microenvironment. (A) NCI-H929, INA6, MM.1S, and RPMI-8226 cells were treated with TAS-116 (1 μM) either alone or in combination with tipifarnib (NCI-H929: 2 μM, INA6: 0.5 μM, MM.1S: 2 μM, RPMI-8226: 2 μM), dabrafenib (NCI-H929: 10 μM, INA6: 2 μM, MM.1S: 10 μM, RPMI-8226: 10 μM), or AZD6244 (NCI-H929: 10 μM, INA6: 2 μM, MM.1S: 20 μM, RPMI-8226: 20 μM) for 48 h. Apoptotic cells were analyzed with flow cytometry using annexin V/PI staining. Each treatment was tested in triplicate wells, and apoptosis was assessed as the percentage of annexin V-positive cells. TAS, TAS-116; Tipi, tipifarnib; Dabra, dabrafenib; AZD, AZD6244. (B) MM.1S and NCI-H929 cells were cultured with TAS-116 (2 μM), AZD6244 (20 μM), or TAS-116 plus AZD6244 for 24 h in the presence or absence of BMSC supernatant. Whole-cell lysates were subjected to western blotting using PARP, caspase 3, and β-actin Abs. FL, full-length; CF, cleaved form; SN, supernatant. (C) MM.1S cells were cultured with TAS-116 (1 μM), AZD6244 (20 μM), or TAS-116 plus AZD6244; and NCI-H929 cells were cultured with TAS-116 (1 μM), AZD6244 (10 μM), or TAS-116 plus AZD6244 for 48 h in the presence or absence of BMSC supernatant. Apoptotic cells were analyzed with flow cytometry using annexin V/PI staining. Each treatment was tested in triplicate wells, and apoptosis was assessed as the percentage of annexin V-positive cells. TAS, TAS-116; AZD, AZD6244; SN, supernatant (*: *P* < 0.05; **: *P* < 0.01).

We next assessed the anti-MM effect of TAS-116 in combination with tipifarnib, dabrafenib, or AZD6244 using the MTT assay. The combination of TAS-116 plus one of these inhibitors induced additive or synergistic cytotoxicity in *RAS*-mutated MM cell lines (Fig 4A, 4B, 4C and 4D and S1–S4 Tables). In addition, annexin V/PI staining showed that TAS-116 significantly enhanced apoptosis induced by these inhibitors in *RAS*-mutated MM cell lines (*P* < 0.01 in each combination treatment versus either monotherapy or control in NCI-H929 and MM.1S cell lines; *P* < 0.05 in each combination treatment versus either monotherapy or control in RPMI-8226 cell line; *P* < 0.01 in dabrafenib and AZD6244 combination treatment versus either monotherapy or control, and *P* < 0.01 in tipifarnib combination treatment versus monotherapy or control in INA6 cell line) (Fig 5A).

We next examined the combination effect on the modulation of the RAS-RAF-MEK-ERK signaling pathway cascades in *RAS*-mutated cell lines using western blot analysis. Treatment with RAS-RAF-MEK-ERK inhibitors induced p-C-Raf activation, and AZD6244 treatment induced significant accumulation of p-MEK1/2, which was significantly inhibited by TAS-116. p-ERK induced by tipifarnib was also inhibited by TAS-116. In NCI-H929 cells, treatment with RAS-RAF-MEK-ERK signaling pathway inhibitors upregulated p-Akt, which was also significantly inhibited by TAS-116. These results suggest that TAS-116 markedly inhibits p-C-Raf, p-MEK1/2, and p-ERK, which were paradoxically activated by these inhibitors. Importantly, cleavage of PARP and caspase 3 was significantly enhanced by treatment with TAS-116 in combination with RAS-RAF-MEK-ERK signaling pathway inhibitors in both cell lines. On the other hand, TAS-116 in combination with dabrafenib paradoxically enhanced p-ERK level, possibly via a feedback mechanism. These results indicate with RAS-RAF-MEK-ERK signaling pathway inhibitors can paradoxically trigger RAS-RAF-MEK-ERK signaling pathway activation via a feedback mechanism, and that combination therapy with TAS-116 blocks this response and induces synergistic cytotoxicity and caspase-dependent apoptosis.

Previous studies have reported that RAS-RAF-MEK-ERK pathway inhibition upregulates Bim, which translocates to mitochondria, releases cytochrome-c, and induces caspase-dependent apoptosis [43]. We therefore hypothesized that TAS-116 could enhance mitochondrial damage-induced apoptosis induced by RAS-RAF-MEK-ERK inhibitors. Importantly, TAS-116 or RAS-RAF-MEK-ERK pathway inhibitors upregulate Bim expression, and the combination further enhances Bim expression (S5A Fig). Moreover, TAS-116 also enhances mitochondrial damage-induced apoptosis by RAS-RAF-MEK-ERK pathway inhibitors (S5B Fig). Taken together, these results suggest that TAS-116 enhances apoptosis induced by RAS-RAF-MEK-ERK pathway inhibitors including dabrafenib via mitochondrial damage in MM cells.

We previously showed that the MEK inhibitor AZD6244 targets both MM cells and the BM microenvironment milieu [44]. Thus, we next hypothesized that TAS-116 enhances the AZD6244-induced anti-MM effect even in the BM microenvironment. For this purpose, we employed BMSC culture supernatant to avoid the effect of these agents on BMSCs, which may downregulate growth/anti-apoptotic soluble factors (ie, IL-6, IGF1). Importantly, western blotting showed that TAS-116 in combination with AZD6244 enhanced cleavage of PARP and caspase 3 in *KRAS*-mutated MM.1S cells, in the presence or absence of BMSC supernatant (Fig 5B). Furthermore, annexin V/PI analysis showed that TAS-116 in combination with AZD6244 enhanced apoptosis compared with TAS-116 or AZD6244 monotherapy (*P* < 0.01, respectively, in both NCI-H929 and MM.1S cell lines), even with BMSC supernatant (*P* < 0.05, respectively, in both cell lines) (Fig 5C). Taken together, these results indicate that the combination of TAS-116 and tipifarnib, dabrafenib, or AZD6244 triggers synergistic anti-MM activity, even in the BM microenvironment.

### TAS-116 induces synergistic cytotoxicity with dabrafenib in the *BRAF*-mutated U266 MM cell line

**Fig 6:**
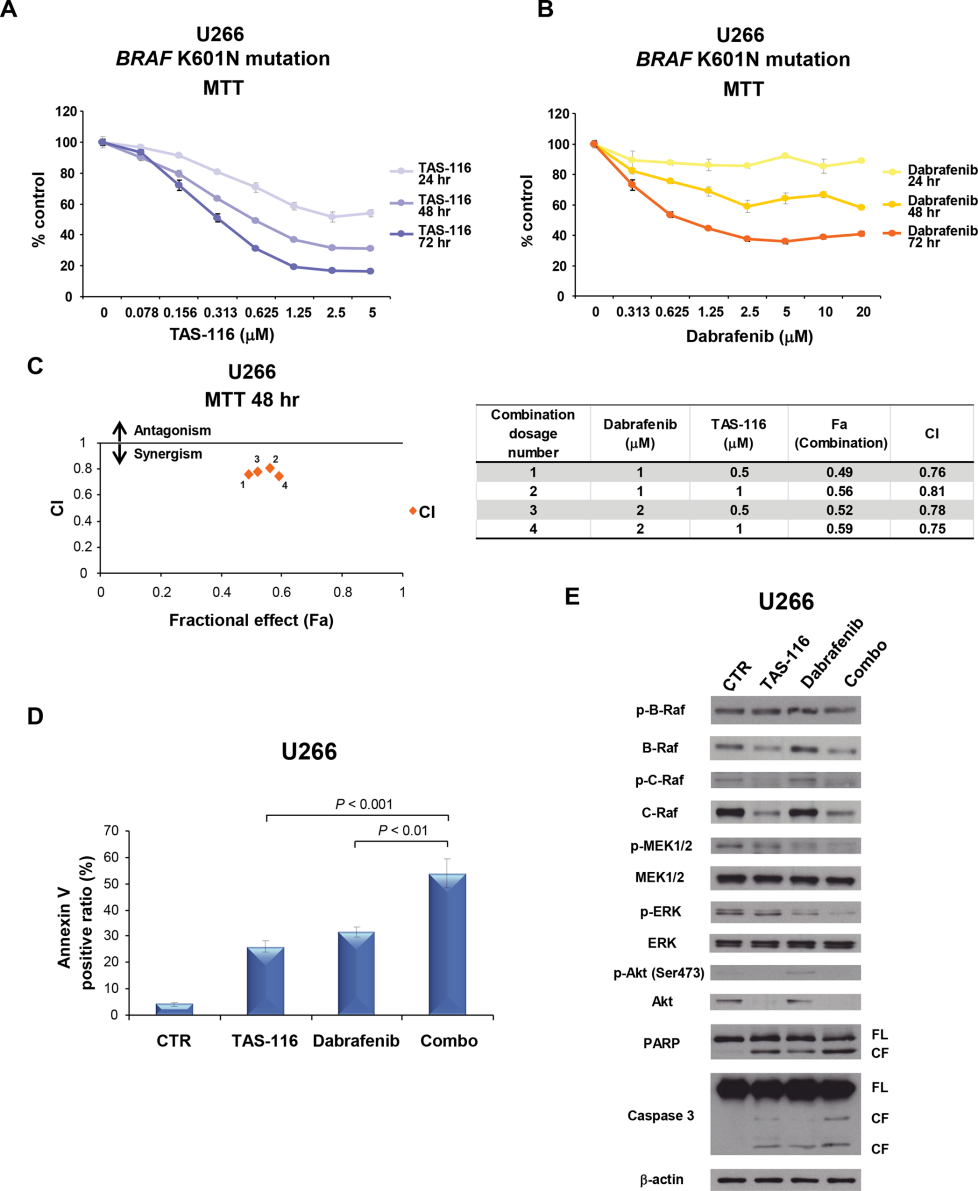
TAS-116 induces synergistic cytotoxicity with dabrafenib in the BRAF-mutated U266 MM cell line. U266 cells were cultured with TAS-116 (0–5 μM) (A) or dabrafenib (0–5 μM) (B) for 24, 48, or 72 h. Cell viability was assessed with the MTT assay of triplicate cultures and expressed as the percentage of the untreated control. Data are the mean ± SD. (C) U266 cells were treated with the indicated concentrations of TAS-116, dabrafenib, or TAS-116 plus dabrafenib for 48 h, and then the viability was analyzed with the MTT assay. Isobologram analysis shows the synergistic cytotoxic effect of TAS-116 and dabrafenib. The graph (left) is derived from the values given in the table (right). The numbers 1–4 in the graph correspond to the combinations shown in the table. CI values < 1.0 indicate synergism; CI = 1.0 indicates an additive effect; and CI > 1.0 indicates antagonism. (D) U266 cells were treated with TAS-116 (0.5 μM), dabrafenib (1 μM), or TAS-116 plus dabrafenib for 48 h. Apoptotic cells were analyzed with flow cytometry using annexin V/PI staining. Each treatment was tested in triplicate wells, and apoptosis was assessed as the percentage of annexin V-positive cells. (E) U266 cells were treated with TAS-116 (1 μM), dabrafenib (2 μM), or TAS-116 plus dabrafenib for 24 h. Whole-cell lysates were subjected to western blotting using p-B-Raf, B-Raf, p-C-Raf, C-Raf, p-MEK1/2, MEK1/2, p-ERK, ERK, p-Akt (S473), Akt, PARP, caspase 3, and β-actin Abs. FL, full-length; CF, cleaved form.

We next examined the cytotoxicity of TAS-116 or dabrafenib in the *BRAF*-mutated (K601N) U266 MM cell line using the MTT assay. TAS-116 or dabrafenib significantly inhibited the viability of U266 MM cells in a dose- and time-dependent manner (Fig 6A and 6B). TAS-116 in combination with dabrafenib induced synergistic cytotoxicity in the U266 MM cell line, evidenced by MTT assay (Fig 6C). In addition, annexin V/PI staining showed that TAS-116 significantly enhanced apoptosis induced by dabrafenib in the U266 MM cell line (*P* < 0.001 in the combination treatment versus TAS-116 monotherapy; *P* < 0.01 in the combination treatment versus dabrafenib monotherapy) (Fig 6D). Western blot analysis showed that TAS-116 markedly inhibited p-C-Raf, which was paradoxically activated by dabrafenib in U266 MM cells (Fig 6E). Importantly, TAS-116 and dabrafenib synergistically inhibited p-MEK1/2 and p-ERK (Fig 6E). Furthermore, cleavage of PARP and caspase 3 was significantly enhanced by TAS-116 in combination with dabrafenib (Fig 6E). Taken together, these results indicate that TAS-116 enhances dabrafenib-induced MM cytotoxicity and apoptosis, even in the *BRAF*-mutated U266 MM cells.

## Discussion

The RAS-RAF-MEK-ERK signaling pathway plays a crucial role in tumorigenesis, cell proliferation, inhibition of apoptosis, and drug resistance [45,46]. Furthermore, *RAS* mutations may play a key role in malignant transformation of clonal plasma cells and MM pathogenesis [23]. In particular, other groups have shown that *NRAS* or *KRAS* mutations confer resistance to conventional or molecularly-targeted therapies in various malignancies, including MM [25,47–49]. Therefore, a novel therapy that overcomes these mechanisms of resistance is needed. In the present study, we focused on the combination of the novel, oral, selective HSP90α/β inhibitor TAS-116 and RAS-RAF-MEK-ERK inhibitors in *RAS*-mutated or *BRAF*-mutated MM cells.

Our previous studies have shown that TAS-116 more potently and significantly targets HSP90 client proteins including C-Raf and MEK1/2 in MM cell lines than 17-AAG [16]. Importantly, we and others have previously reported that *NRAS* mutation significantly reduces MM sensitivity to single-agent bortezomib therapy [25]. Patients with *RAS* mutations also have significantly shorter progression-free and overall survival than patients without this mutation [24]. In this study, we demonstrated that TAS-116 potently induces cytotoxicity and significantly inhibits RAS-RAF-MEK-ERK pathway client proteins in *NRAS-* or *KRAS-*mutated MM cell lines. These results suggest that TAS-116 potently overcomes the resistance mechanism induced by *RAS* mutations.

The RAS-RAF-MEK-ERK pathway is activated in a variety of tumors, including MM [20,50]; therefore, RAS-RAF-MEK-ERK pathway inhibitors are promising therapeutic options for cancer therapy. However, inhibition of the RAS-RAF-MEK-ERK pathway may be mainly cytostatic [20] and show significant efficacy primarily at an early stage of treatment, since resistance develops due to activation of other signaling pathways via feedback mechanisms or paradoxical activation mechanisms [51]. Therefore, there is a need to combine inhibitors of the RAS-RAF-MEK-ERK with a drug to target and overcome these resistance mechanisms. In the present study, we show that TAS-116 triggers significant synergistic anti-MM effects when used in combination with a RAS-RAF-MEK-ERK pathway inhibitor. Importantly, TAS-116 inhibited the paradoxical activation of p-C-Raf, p-MEK, or p-ERK triggered by these inhibitors, and markedly enhanced RAS-RAF-MEK-ERK pathway inhibitor-induced apoptosis. Moreover, this enhanced apoptosis in *RAS*-mutated MM triggered by combination treatment was observed even in the presence of BMSCs. Taken together, our results provide the rationale for a novel treatment strategy combining selective HSP90α/β inhibitor and RAS-RAF-MEK-ERK signaling pathway inhibitors to improve patient outcome in *RAS*-mutated MM.

Other groups have shown that dabrafenib shows significant *in vitro* and *in vivo* anti-tumor effects in malignant melanoma with the *BRAF V600E* mutation. However, the therapeutic effects are often temporary due to development of several resistance mechanisms [40]. One mechanism is ERK signaling resistant to treatment with RAF inhibitors due to increased RAF dimers in cells, and another involves bypassing the dependence of the tumor on mutant RAF. Moreover, tumors with wild-type BRAF, including those with *RAS* mutation, exhibit a paradoxical activation of ERK signaling when treated with these drugs [28,40,41]. Importantly, many proteins in the RAS-RAF-MEK-ERK pathway are client proteins of HSP90; therefore, a combination of TAS-116 and a RAS-RAF-MEK-ERK pathway inhibitor may abrogate the feedback mechanisms that promote resistance to dabrafenib. In the present study, we focused on whether TAS-116 induces a synergistic effect or overcomes these resistance mechanisms when combined with dabrafenib to treat MM cells with *RAS* and *BRAF* mutations. We show that TAS-116 induced synergistic anti-MM cytotoxicity and enhanced dabrafenib-induced apoptosis in *RAS*-mutated MM cell lines. However, TAS-116 paradoxically enhanced p-ERK when combined with dabrafenib,suggesting that MEK-ERK signaling may not contribute to MM cell growth inhibition triggered by this combination treatment. Because HSP90 inhibitors block multiple pathways crucial to MM survival [7,9,20], the combination of TAS-116 and dabrafenib enhances the anti-MM effect in *RAS*-mutated and wild-type *BRAF* MM cells via a mechanism other than ERK pathway inhibition.

Whole-genome sequencing data have recently revealed that a subset of patients carry an activating mutation (V600E) in the BRAF kinase [52]. In addition, other groups recently reported a significant response to the BRAF inhibitor vemurafenib in *BRAF V600E*-mutated MM patients in the clinical setting [52,53]. In the laboratory setting, BRAF inhibition also significantly downregulates MEK-ERK pathway activity in *BRAF K601N*-mutated U266 MM cells [54]. Therefore, these data indicate that *BRAF* mutation is a promising target for MM therapy. However, several mechanisms of resistance to RAF inhibitors have been proposed [40], and several combinatorial therapy approaches have already been pursued to improve the outcome of patients with BRAF V600E melanomas treated with BRAF inhibitors [40]. In the present study, we focused on the significance of a combination of TAS-116 and dabrafenib. TAS-116 triggered significant synergistic anti-MM effects and enhanced apoptosis induced by dabrafenib in the U266 MM cell line. Importantly, TAS-116 enhanced the inhibition of p-MEK1/2 or p-ERK induced by dabrafenib. Moreover, TAS-116 markedly inhibited p-C-Raf or p-Akt, which were paradoxically activated by dabrafenib in the U266 MM cell line. Taken together, these results suggest that the combination of TAS-116 and dabrafenib has a synergistic anti-MM effect, even in *BRAF*-mutated MM cells.

In conclusion, TAS-116 in combination with a RAS-RAF-MEK-ERK signaling pathway inhibitor shows significant synergistic anti-MM effects in *RAS*- or *BRAF*-mutated MM cell lines *in vitro*, providing the framework for its clinical evaluation to improve the outcome of this subset of patients with MM.

## Supporting Information

S1 Fig TAS-116 is a novel, oral, selective HSP90α/β inhibitor. Chemical structure of TAS-116.
(EPS)

S2 Fig RAS knockdown induces cell cycle modulation and enhances cytotoxicity induced by bortezomib and doxorubicin in *RAS*-mutated MM cells. NCI-H929 and KMS11 cells were transiently transfected with non-targeting or *NRAS* siRNA and then treated with or without bortezomib (0–10 nM) or doxorubicin (0–400 nM) for 48 h. In each case, cell viability was assessed with the MTT assay of triplicate cultures and expressed as the percentage of the untreated control. Data are the mean ± SD.
(EPS)

S3 Fig TAS-116 triggered a decrease in mitochondria transmembrane potential. MM.1S cells were treated with or without 2 μM TAS-116 for 4, 8, or 24 h, with addition of MitoCapture reagent (MitoCapture Apoptosis Detection kit, Calbiochem) for the last 20 minutes, followed by flow cytometric analysis.
(EPS)

S4 Fig The RAF inhibitor dabrafenib induces paradoxical activation of ERK signaling in *RAS*-mutated MM cells. NCI-H929 and RPMI-8226 cells were treated with the indicated concentrations of dabrafenib for 24 h. Whole-cell lysates were subjected to western blotting using p-B-Raf, B-Raf, p-C-Raf, C-Raf, p-MEK1/2, MEK1/2, p-ERK, ERK, and β-actin Abs.
(EPS)

S5 Fig TAS-116 enhances mitochondrial apoptosis induced by RAS-RAF-MEK-ERK pathway inhibitors. (A) NCI-H929 and RPMI-8226 cells were treated with the indicated concentrations of TAS-116 either alone or in combination with tipifarnib, dabrafenib, or AZD6244 for 24 h. Whole-cell lysates were subjected to western blotting using Bim and α-tubulin Abs. (B) NCI-H929 cells were treated with the indicated concentrations of TAS-116 either alone or in combination with tipifarnib, dabrafenib, or AZD6244 for 12 h, with addition of MitoCapture reagent (MitoCapture Apoptosis Detection kit, Calbiochem) for the last 20 minutes, followed by flow cytometric analysis. MFI, mean fluorescent intensity.
(EPS)

S1 Table TAS-116 combination indices (CI) with tipifarnib, dabrafenib, or AZD6244 in NCI-H929 cells. The tables correspond to the Fa and CI values in Fig 4A. CI was calculated using CompuSyn software.
(EPS)

S2 Table TAS-116 combination indices (CI) with tipifarnib, dabrafenib, or AZD6244 in INA6 cells. The tables correspond to the Fa and CI values in Fig 4B. CI was calculated using CompuSyn software.
(EPS)

S3 Table TAS-116 combination indices (CI) with tipifarnib, dabrafenib, or AZD6244 in RPMI-8226 cells. The tables correspond to the Fa and CI values in Fig 4C. CI was calculated using CompuSyn software.
(EPS)

S4 Table TAS-116 combination indices (CI) with tipifarnib, dabrafenib, or AZD6244 in MM.1S cells. The tables correspond to the Fa and CI values in Fig 4D. CI was calculated using CompuSyn software.
(EPS)
